# The effects of coconut oil intake on metabolic disorders and fatty liver disease in mice

**DOI:** 10.1017/jns.2025.10032

**Published:** 2025-08-26

**Authors:** Madoka Sumi, Yuka Hasegawa, Tomoyuki Matsuyama, Tomoki Miyoshi, Hanako Nakajima, Takuro Okamura, Naoko Nakanishi, Ryoichi Sasano, Masahide Hamaguchi, Michiaki Fukui

**Affiliations:** 1 Department of Endocrinology and Metabolism, Graduate School of Medical Science, Kyoto Prefectural University of Medicine, Kyoto, Japan; 2 Department of Diabetes and Endocrinology, Kyoto Okamoto Memorial Hospital, Kyoto, Japan; 3 AiSTI Science Co., Ltd., Wakayama, Japan

**Keywords:** Fatty liver disease, Gas chromatography-mass spectrometry, Medium-chain fatty acids, Coconut oil, ALT, Alanine aminotransferase, AST, Aspartate aminotransferase, AUC, Area under the curve, Ct, Threshold cycle, CVD, Cardiovascular disease, GC/MS, Gas chromatography-mass spectrometry, iPGTT, Intraperitoneal glucose tolerance test, ITT, Insulin tolerance test, LCFAs, Long-chain fatty acids, MASLD, Metabolic dysfunction-associated steatotic liver disease, MCFAs, Medium-chain fatty acids, MCTs, Medium-chain triglycerides, NAFLD, Non-alcoholic fatty liver disease, NAS, NAFLD activity score, NEFA, Non-esterified fatty acid, RT-PCR, Reverse transcription-polymerase chain reaction, SD, Standard deviation, TG, Triglyceride, T-Cho, Total cholesterol

## Abstract

High-fat diets are closely implicated in the pathogenesis of chronic conditions, including obesity and hepatic steatosis. Recently, coconut oil, which is rich in medium-chain fatty acids, has attracted significant attention for its potential anti-obesity and anti-inflammatory properties. This study aimed to evaluate the effects of medium-chain fatty acids derived from coconut oil on metabolic disorders, particularly fatty liver, using a mouse model established by a high-fat diet. C57BL/6J mice were assigned to either the lard diet group or the coconut oil diet group and fed for 12 weeks. Glucose tolerance was assessed, and biochemical parameters, liver histology, and gene expression in the liver were analysed. Additionally, the concentrations of medium-chain fatty acids within the liver were determined through gas chromatography-mass spectrometry analysis. Mice fed a coconut oil diet exhibited suppressed weight gain and improved glucose tolerance compared to mice fed a lard diet. Furthermore, the coconut oil diet resulted in reduced hepatic fat accumulation, decreased expression levels of genes implicated in inflammation and lipid metabolism within the liver, and higher concentrations of medium-chain fatty acids in the liver. Coconut oil may contribute to the suppression of hepatic fat accumulation in the liver and the prevention of non-alcoholic fatty liver disease/metabolic dysfunction-associated steatotic liver disease by increasing the levels of medium-chain fatty acids in the liver and suppressing the expression of genes implicated in inflammation and lipid metabolism.

## Introduction

Lipids are one of the three major macronutrients and are essential components of the diet; however, high-fat diets have been suggested to increase energy intake and promote obesity.^(1)^ Obesity is strongly associated with chronic conditions such as cardiovascular diseases (CVD), diabetes, and non-alcoholic fatty liver disease (NAFLD)/metabolic dysfunction-associated steatotic liver disease (MASLD).^(2–7)^

Lipids are classified into saturated and unsaturated fatty acids, and excessive dietary intake of saturated fatty acids has been associated with an increased risk of CVD and hepatic fat accumulation, thereby exerting adverse health effects.^(8,9)^ Fatty acids are categorised based on carbon chain length into short-chain fatty acids, medium-chain fatty acids (MCFAs), and long-chain fatty acids (LCFAs). Representative LCFAs include palmitic acid (C16:0), stearic acid (C18:0), and oleic acid (C18:1). Previous studies indicate that LCFAs pose a higher CVD risk than MCFAs,^(10)^ particularly palmitic acid (C16:0), which has been identified as a major contributor to atherosclerosis development.^(11,12)^ Additionally, another study demonstrated that LCFAs are taken up by cells primarily through facilitated transport (>90%), which has been reported to be upregulated in adipocytes from obese rats, mice, and humans.^(13)^

In contrast, studies on MCFAs remain relatively limited. MCFAs exhibit distinct characteristics compared to LCFAs, including higher ketogenic potential, and differences in digestion and metabolic pathways.^(14)^ MCFAs are directly transported to the liver via the portal vein and rapidly utilised for energy through β-oxidation, resulting in a lower propensity for storage as body fat.^(15)^ A previous study using chick embryos and HepG2 hepatocytes demonstrated that MCFAs such as caproic acid (C6) and caprylic acid (C8) play a crucial role in reducing the expression and activity of fatty acid synthase, a key enzyme involved in de novo lipogenesis.^(16)^ These findings suggest that MCFAs exert protective effects on lipid metabolism derangement and inflammation in the liver.

Recently, the anti-obesity, antioxidant, and anti-inflammatory effects of coconut oil, which is rich in MCFAs, have gained significant attention.^(17)^ This study aims to elucidate the effects of MCFAs in coconut oil on metabolism using a high-fat diet-induced mouse model through metabolomic analysis via gas chromatography-mass spectrometry (GC/MS), clarify the mechanisms by which MCFAs suppress fatty liver, and propose the utility of nutritional interventions for fatty liver.

## Materials and methods

### Mice

All experimental protocols were rigorously examined and authorised by the Animal Research Committee at Kyoto Prefectural University of Medicine, Japan (Approval No. M2024-77). Eight-week-old male C57BL/6J (wild-type) mice (*n* = 16) were procured from Shimizu Laboratory Supplies (Kyoto, Japan) and maintained in a meticulously controlled, pathogen-free environment. The mice were housed in cages with four mice per cage. The mice were fed two distinct high-fat diets for 12 weeks, starting at 8 weeks of age (*n* = 8 per group). The experimental diets consisted of a lard-based high-fat diet (D12492, Research Diets, Inc., New Brunswick, NJ, USA) and a coconut oil-based high-fat diet (D00071501, Research Diets). Both diets were formulated on a calorie basis, providing 60% of total energy from fat (from lard or hydrogenated coconut oil, respectively), 20% from protein, and 20% from carbohydrate, with an energy density of 524 kcal per 100 g. While lard and hydrogenated coconut oil served as the principal fat sources, both diets also contained 25 g/kg of soybean oil to ensure adequate intake of essential fatty acids, including linoleic acid (n-6) and α-linolenic acid (n-3). These formulations are widely utilised in metabolic research and are designed to meet the minimum nutritional requirements of laboratory rodents. The detailed composition of each diet is shown in Table 1, and a comparative analysis of the fatty acid profiles is presented in Table 2. We acknowledge that this dietary composition may not reflect typical human intake patterns; therefore, future studies will investigate partial substitution models to improve translational relevance. In the present study, a normal diet group was not included due to the substantial differences in caloric density between standard rodent chow and the high-fat diets used. Inclusion of a normal diet group would have resulted in unequal total caloric intake, thereby complicating direct metabolic comparisons with the experimental groups. To ensure consistency in energy intake across groups, we employed a pair-feeding protocol between the lard and coconut oil groups, as both diets provided equivalent caloric content per unit weight. Although the absence of a normal diet group limits the ability to assess absolute levels of obesity, this design allowed for a controlled evaluation of the differential effects of fat type under isocaloric conditions. We acknowledge this limitation and intend to incorporate a normal diet group in future studies to improve the translational relevance and provide a more comprehensive understanding of dietary fat-induced obesity and metabolic dysfunction. Body weight was measured once a week, and upon reaching 20 weeks of age, they were euthanized following an overnight fast through the administration of a combination of 5.0 mg/kg butorphanol, 4.0 mg/kg midazolam, and 0.3 mg/kg medetomidine.^(18)^ All investigators were aware of the experimental conditions, and blinding was not implemented. No mice were excluded from the analysis in any of the experimental groups.

**Table 1.** Detailed composition of experimental diets (g/kg)

Ingredient	D12492 (lard diet)	D00071501 (coconut oil diet)
Casein	200	200
L-Cystine	3	3
Maltodextrin 10	125	125
Sucrose	72.8	72.8
Cellulose	50	50
Soybean Oil	25	25
Lard	245	0
Hydrogenated Coconut Oil	0	245
Mineral Mix (S10026B)	50	50
Vitamin Mix (V10001C)	1	1
Choline Bitartrate	2	2
FD&C Blue #1	0.05	0.05
Total	773.85	773.85
Energy (kcal/100g)	524	524
Macronutrient Ratio (% kcal)	Fat 60%, Protein 20%, Carbohydrate 20%	Same as D12492

Values are expressed in grams per kilogram of diet (g/kg).

**Table 2.** Fatty acid composition of dietary fats used in the experimental diets

	Lard	Coconut oil
	(%)	(%)
Saturated	40	90
Caprylic acid (C8:0)	–	8
Capric acid (C10:0)	–	7
Lauric acid (C12:0)	–	48
Myristic acid (C14:0)	2	16
Palmitic acid (C16:0)	27	9
Stearic acid (C18:0)	11	2
Unsaturated	59	9
Oleic acid (C18:1(n-9))	44	7
Linoleic acid (C18:2(n-6))	11	2
Linolenic acid (C18:3)	–	–
Palmitoleic acid (C16:1(n-7))	4	–
Other	1	1

Values are expressed as percentage by weight (w/w) of each fatty acid in the total fat content.

### Sample size calculation

The required sample size was determined using a power analysis for a two-sample independent *t*-test. The NAFLD Activity Score (NAS) was used as the primary outcome measure. The mean and standard deviation for Group A were 3.5 and 1.29, respectively, while those for Group B were 1.0 and 1.15. The effect size (Cohen’s d) was calculated using the pooled standard deviation. With a significance level (α) of 0.05 and a power (1−β) of 0.8, the minimum required sample size per group was determined to be 6 using the solve power function from the statsmodels Python package. Although this sample size was targeted, the actual number of animals varied among experiments due to differences in tissue availability and adherence to animal welfare guidelines aimed at minimising animal use. For gene expression analyses, *n* = 3 was used with technical replicates, and consistent trends were observed across biological replicates. We acknowledge the limitation of the small sample size and plan to increase the number of biological replicates in future studies.

### Analytical procedures and glucose and insulin tolerance tests

In 19-week-old mice, an intraperitoneal glucose tolerance test (iPGTT) was conducted after a 16-h fasting period, using a glucose dose of 2 g/kg body weight. Blood glucose levels were measured at designated time points by obtaining a drop of blood and analysing it with a glucometer. Furthermore, an insulin tolerance test (ITT) was performed after a 4-h fasting period, with an insulin dose of 0.5 U/kg body weight. The area under the curve (AUC) was calculated for both iPGTT and ITT results to assess glucose and insulin responses.

### Biochemistry

Blood samples were obtained via cardiac puncture from fasted mice at the time of sacrifice, and the concentrations of aspartate aminotransferase (AST), alanine aminotransferase (ALT), total cholesterol (T-Cho), triglyceride (TG), and non-esterified fatty acid (NEFA) were assessed. Biochemical analyses were outsourced to Oriental Yeast Co., Ltd. (Itabashi, Tokyo, Japan). The analytical methods and reagents used for each parameter were as follows:

AST and ALT levels were measured by a colorimetric method standardised by the Japan Society of Clinical Chemistry (JSCC), using commercial kits (L-Type Wako AST/ALT·J2; FUJIFILM Wako Pure Chemical Corporation, Osaka, Japan). T-Cho and TG levels were determined enzymatically using L-Type Wako CHO·M and TG·M kits (FUJIFILM Wako), following the manufacturer’s instructions. NEFA levels were quantified enzymatically using the NEFA-HR kit for automated analyzers (FUJIFILM Wako), in accordance with the manufacturer’s protocol.

### Liver histology

Liver tissue was collected and immediately fixed in 10% buffered formaldehyde before being embedded in paraffin. Thin sections of the liver, cut to 4 μm, were prepared and stained with haematoxylin and eosin. A stock solution of Oil Red-O in isopropanol (0.25 g/100 mL) was prepared and subjected to heating at 100°C for 10 min. The hepatic sections were then treated with 4% paraformaldehyde for 30 min, followed by rinsing with PBS. A 60% Oil Red-O working solution was created by diluting the stock solution with deionised water, and the sections were incubated in this solution for 30 min. After staining, the sections were rinsed with PBS until the background was rendered clear. Images of the liver sections were acquired using a BZ-X710 fluorescence microscope (Keyence). Furthermore, to evaluate the severity of NAFLD, NAS^(19)^ was calculated. This score is a widely recognised method for assessing the severity of NASH and tracking the progression or improvement of NAFLD. The scoring system encompassed 14 histological parameters, four of which were assessed semi-quantitatively: steatosis (0–3), lobular inflammation (0–2), hepatocellular ballooning (0–2), and fibrosis (0–4).

### Gene expression analysis in the liver

Livers from mice subjected to a 16-h fasting period were excised and immediately cryopreserved in cryogenic liquid nitrogen. The tissues were homogenised in ice-cold QIAzol Lysis reagent (Qiagen) at 4000 rpm for 2 min using a ball mill, and total RNA was subsequently isolated following the manufacturer’s protocol. RNA concentration and integrity were assessed using the Qubit RNA Assay (Invitrogen). Reverse transcription of 0.5 μg of total RNA to first-strand cDNA was performed using the High-Capacity cDNA Reverse Transcription Kit (Applied Biosystems), following the manufacturer’s instructions. The mRNA expression levels of *Tnfa*, *Il6*, *Il1b*, *Scd1*, *Elovl6*, and *Fasn* in liver tissues were quantified through real-time reverse transcription-polymerase chain reaction (RT-PCR) utilising TaqMan Fast Advanced Master Mix (Applied Biosystems). The PCR protocol was as follows: an initial phase of 2 min at 50°C and 20 sec at 95°C was followed by 40 amplification cycles consisting of 1 sec at 95°C and 20 sec at 60°C. The relative expression levels of the target genes were normalised to the threshold cycle (Ct) values of *Gapdh* and quantified utilising the comparative threshold cycle 2^–ΔΔCt^ method. The expression signals from the lard-fed group were designated a relative value of 1.0. Gene expression was assessed using liver samples from six mice per group, with each sample analysed in triplicate.

### Measurement of MCFAs levels in the liver

The composition of MCFAs in the liver was analysed utilising GC/MS with an Agilent 7890B/7000D system (Agilent Technologies, Santa Clara, CA, USA). Briefly, 5 mg of liver sample was subjected to methylation using a fatty acid methylation kit (Nacalai Tesque, Kyoto, Japan). The resulting samples were injected into a capillary column, Vf-5 ms (30 m × 0.25 mm [inner diameter] × 0.25 μm [film thickness]; Agilent Technologies). The column temperature was initially held at 80 °C for 3 min, after which it was incrementally raised at a rate of 25 °C/min until it reached 190 °C. It was then further raised at a rate of 3°C/min to 220°C, followed by an increase at a rate of 15°C/min to 310°C, where it was maintained for 4.6 min. The injection was performed in split mode with a 5:1 split ratio. Fatty acid methyl esters were identified via selected ion monitoring, and data analysis was performed by normalising the peak intensities against the C17:0 internal standard.

### Statistical analysis

Data analysis was performed using JMP software version 17.2 (SAS, Cary, NC, USA). Paired *t*-tests were conducted to assess the differences between the groups. A *P*-value of less than 0.05 was regarded as statistically significant. Graphical representations were generated utilising GraphPad Prism software (version 10.2.2; San Diego, CA, USA).

## Results

### Coconut oil suppressed weight gain and improved glucose tolerance compared to lard

**Figure 1.**
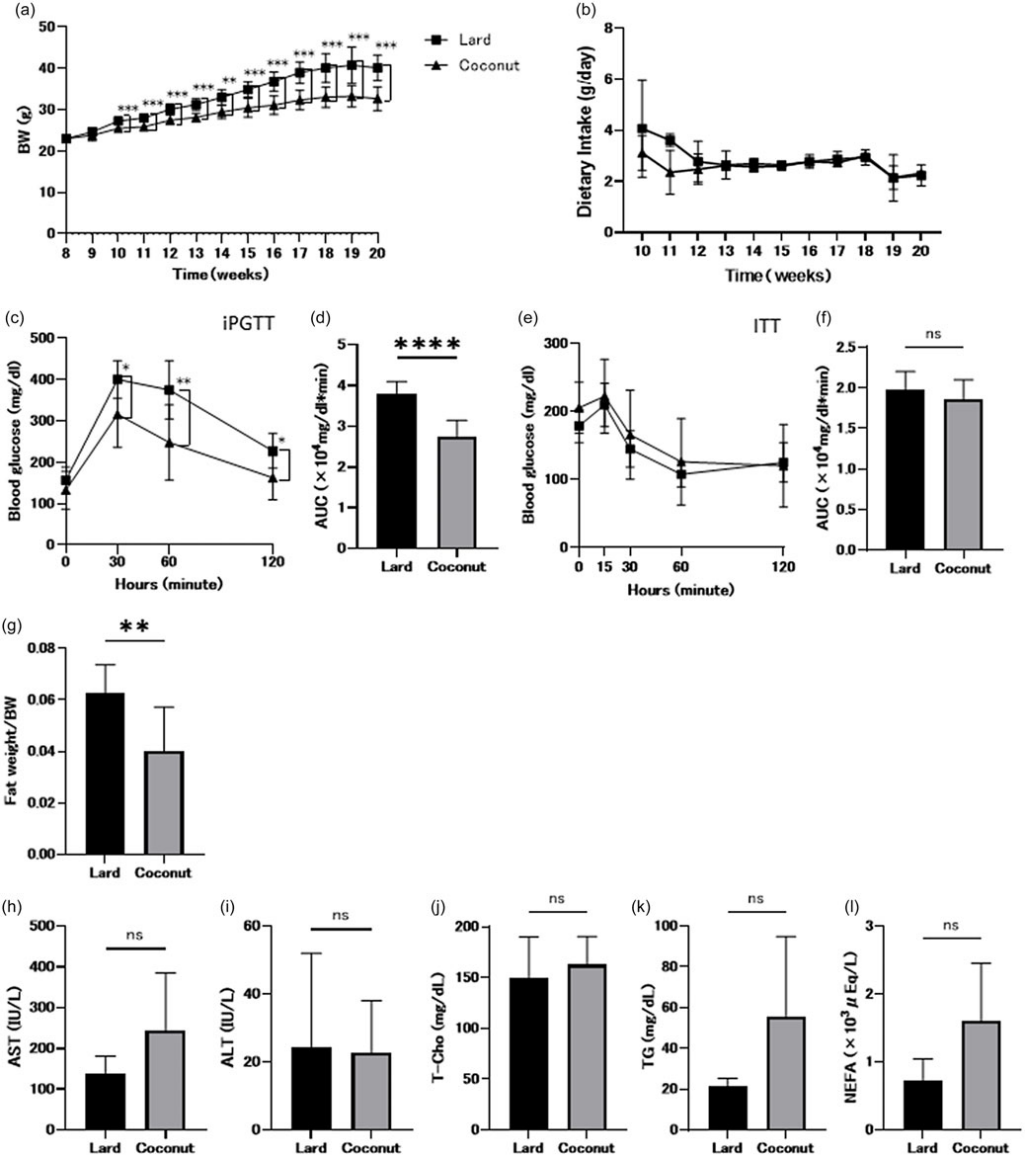
Body weight and food intake in mice fed with lard or coconut oil from 8 weeks to 20 weeks of age, iPGTT and ITT at 19 weeks of age, and fat weight and blood test at 20 weeks of age. (a) Changes in body weight (*n* = 8). (b) Changes in the intake of food (*n* = 8). (c and d) Results of iPGTT (2 g/kg body weight) for 19-week-old mice and AUC analysis (*n* = 8). (e and f) Results of ITT (0.5 U/kg body weight) for 19-week-old mice and AUC analysis (*n* = 8). (g) Relative fat weight (*n* = 8). (h–l) Serum aspartate aminotransferase (AST), alanine aminotransferase (ALT), total cholesterol (T-Cho), triglycerides (TG), and non-esterified fatty acids (NEFA) levels (*n* = 5). Data are represented as the mean ± SD values. *P < 0.05, **P < 0.01, ***P < 0.001, and ****P < 0.0001. iPGTT, intraperitoneal glucose tolerance test; AUC, area under the curve; ITT, insulin tolerance test; SD, standard deviation.

From 8 to 20 weeks of age, body weight, food intake, iPGTT, and ITT were assessed in mice fed either a lard-based or coconut oil-based diet. The body weight of the coconut oil-fed group was significantly lower than that of the lard-fed group (Figure 1a). However, no statistically significant difference in food intake was detected between the two groups (Figure 1b). Glucose tolerance was evaluated through iPGTT and ITT. Glucose tolerance worsened in the lard diet group compared to the coconut oil diet group in iPGTT (Figure 1c and d), but it did not worsen in either group in ITT (Figure 1e and f). Fat weight was measured in mice from the lard diet and coconut oil diet groups at 20 weeks of age. The relative fat weight was found to be greater in the lard diet group (Figure 1g).

### Coconut oil did not induce any changes in serum liver enzyme levels or lipid profiles

Next, we examined the serum concentrations of hepatic enzymes and lipid profiles. No significant differences were observed in the serum levels of AST, ALT, T-Cho, TG, and NEFA between the lard diet group and the coconut oil diet group (Figure 1h–l).

### Coconut oil resulted in less hepatic fat accumulation compared to lard

**Figure 2.**
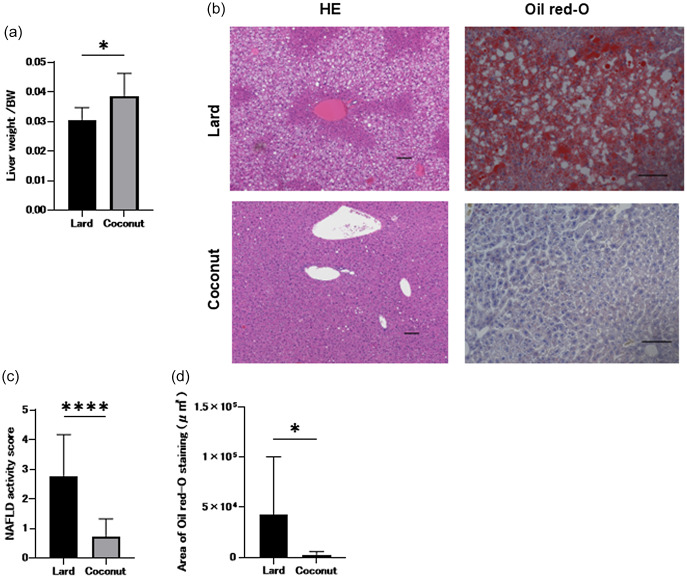
Histological evaluation of liver. (a) Relative liver weight (*n* = 8). (b) Representative images of haematoxylin & eosin (HE)- and Oil red-O-stained liver sections. Liver tissue was collected at 20 weeks of age. The scale bars show 100 μm. (c) Nonalcoholic fatty liver disease (NAFLD) activity scores (*n* = 6). (d) Area of Oil red-O-stained region (*n* = 6). Data are presented as mean ± SD values. *P < 0.05, ***P < 0.001 and ****P < 0.0001.

While the absolute liver weight did not differ significantly between groups, the liver weight-to-body weight ratio was significantly higher in the coconut oil group (Figure 2a). Representative histological micrographs of the liver are presented in Figure 2b. When comparing the lard diet group with the coconut oil diet group, the NAFLD activity score and the extent of Oil Red-O staining were significantly elevated in the lard diet group (Figure 2c and d).

### Coconut oil resulted in a higher concentration of MCFAs in the liver compared to lard

The concentration of MCFAs in the liver was quantified using a GC/MS system, and the results are summarised in Table 3. Levels of caprylic acid (C8:0), capric acid (C10:0), lauric acid (C12:0), myristoleic acid (C14:1), myristic acid (C14:0) were higher in the coconut oil diet group. In contrast, gamma-linolenic acid (C18:3(n-6)) and linoleic acid (C18:2(n-6)) were higher in the lard diet group. The concentrations of palmitic acid (C16:0) and oleic acid (C18:1(n-9)) tended to be lower in the coconut oil diet group compared to the lard diet group.

**Table 3.** The concentration of medium-chain fatty acids in the liver

	Lard (μg/mg)	Coconut (μg/mg)	*P*-value
Caprylic acid (C8:0)	0.00 (0.00)	0.01 (0.00)	0.001
Capric acid (C10:0)	0.00 (0.00)	0.03 (0.02)	0.001
Lauric acid (C12:0)	0.01 (0.00)	0.84 (0.37)	< 0.001
Myristoleic acid (C14:1)	0.01 (0.00)	0.19 (0.06)	< 0.001
Myristic acid (C14:0)	0.32 (0.18)	2.12 (0.62)	< 0.001
Pentadecanoic acid (C15:0)	0.07 (0.03)	0.06 (0.01)	0.650
Palmitic acid (C16:0)	16.40 (7.80)	13.00 (3.80)	0.300
Stearic acid (C18:0)	3.79 (0.40)	3.54 (0.16)	0.130
Oleic acid (C18:1(n-9))	21.00 (11.50)	13.20 (5.10)	0.110
Linoleic acid (C18:2(n-6))	12.10 (5.10)	4.94 (0.67)	0.002
gamma-linolenic acid (C18:3(n-6))	0.35 (0.13)	0.10 (0.02)	<0.001
Arachidic acid (C20:0)	0.21 (0.09)	0.20 (0.06)	0.900

Data are expressed as mean standard deviation (SD). Student’s paired *t*-test was conducted between the two groups.

### Coconut oil exhibited lower gene expression associated with inflammation and lipid metabolism in the liver compared to lard

**Figure 3.**
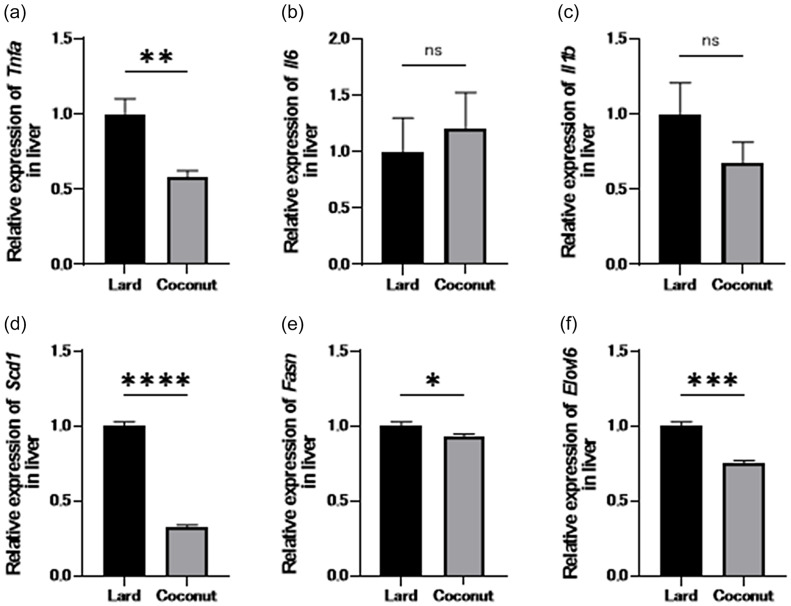
Genes expression of inflammation and fat metabolism in the liver. Relative mRNA expression of (a) *Tnfa*, (b) *Il6*, (c) *Il1b*, (d) *Scd1,* (e) *Fasn*, and (f) *Elovl6* in the liver normalised to the expression of *Gapdh* (*n* = 3). Data are represented as the mean ± SD values. *P < 0.05, **P < 0.01, ***P < 0.001, and ****P < 0.0001.

Expression of genes implicated in inflammation and lipid metabolism in the liver was assessed and compared between the two groups using RT-PCR. The expression levels of *Tnfa*, *Scd1*, *Fasn*, and *Elovl6* were significantly lower in the coconut oil diet group compared to the lard diet group. Nevertheless, no statistically significant differences were observed in the expression levels of *Il6* and *Il1b* between the two groups (Figure 3a–f).

## Discussion

In this study, mice fed a coconut oil diet exhibited reduced body weight gain and improved glucose tolerance compared to those fed a lard diet. Furthermore, the mice in the coconut oil diet group exhibited reduced hepatic fat accumulation, decreased expression of genes implicated in inflammation and lipid metabolism, and an increase in the concentrations of MCFAs in the liver.

Recent studies highlight the health benefits of coconut oil, including its antioxidant, anti-obesity, and anti-inflammatory effects.^(17)^ Interest in MCFAs found in plant oils has also surged in recent years. According to previous studies, coconut oil and MCFAs have been shown to reduce the size and weight of adipose tissue and decrease lipid accumulation in the liver. These findings suggest that supplementation with coconut oil and MCFAs may play a potential role in improving NAFLD/MASLD.^(20,21)^

In our study, although no differences in food intake were observed between the coconut oil diet group and the lard diet group, the coconut oil diet group exhibited suppressed weight gain compared to the lard diet group. Coconut oil is composed of approximately 90% saturated fatty acids, with about 60% of its fatty acid composition comprising MCFAs.^(22)^ Previous studies have reported that coconut oil and MCFAs suppress weight gain induced by a high-fat diet by reducing body fat, which aligns with the findings of the current study.^(21)^ In this study, improved glucose tolerance was observed in the coconut oil diet group. Previous studies have shown that MCFAs and coconut oil enhance insulin sensitivity,^(21)^ and that capric acid (C10:0) improves insulin sensitivity and prevents obesity by regulating glucose homeostasis through glucagon-like peptide-1 secretion mediated by GPR84.^(23)^ In the current study, we confirmed an increase in capric acid, suggested that these mechanisms might be related; however, further research is needed to elucidate the underlying pathways. Additionally, Nonaka *et al.* reported that replacing lard with various medium-chain triglycerides (MCTs), the triglyceride forms of MCFAs (caprylic triglyceride (TriC8), capric triglyceride (TriC10), and lauric triglyceride (TriC12)), in the diet did not lead to significant differences in NEFA concentrations. However, the T-Cho concentrations were lower in all MCT diet groups in comparison to the lard diet group, and the TG concentration was significantly higher in the TriC12 diet group than in the lard diet group.^(23)^ In our study, no statistically significant differences were observed between the lard and coconut oil diet groups in terms of TG, T-Cho, or NEFA levels. The discrepancy in TG and T-Cho results compared with previous reports remains unclear; however, differences in feeding duration and the composition of the fat diet may be contributing factors. Another previous study reported that 2.5–3.5-month-old Wistar rats, when orally administered MCT or lard for 28 consecutive days, showed no significant differences in AST and ALT activity between the two groups, which is consistent with the findings of our study.^(24)^

In this study, although absolute liver weight was significantly higher in the coconut oil group than in the lard group, hepatic lipid accumulation was lower in the former. Previous research has shown that TriC8 increases liver weight, suggesting that C8:0 metabolism may contribute to hepatic hypertrophy, although the exact mechanisms remain unclear.^(23)^ In contrast, other studies have reported that progressive hepatic inflammation is associated with a reduction in liver size in fatty liver disease.^(25)^ Therefore, the lower liver weight observed in the lard group may be partly attributable to inflammation-induced hepatic atrophy.

Consistent with previous findings by Zicker *et al.*, who reported that virgin coconut oil ameliorates fatty liver, the coconut oil group in our study showed significantly lower NAFLD activity scores and Oil Red O-stained areas.^(20)^ Moreover, hepatic concentrations of MCFAs, including caprylic acid (C8:0), capric acid (C10:0), and lauric acid (C12:0), were significantly higher in the coconut oil group. A prior study using female Wistar rats demonstrated that high intake of virgin coconut oil increased hepatic and adipose tissue levels of capric acid (C10:0) and myristic acid (C14:0) compared to soybean oil.^(20)^ Similarly, our results indicate that coconut oil more effectively elevated hepatic MCFA levels than a lard-based diet, suggesting broader applicability of this effect.

In addition, hepatic levels of myristoleic acid (C14:1) were significantly elevated in the coconut oil group (Table 3). Myristoleic acid has been reported to promote the activation of brown adipose tissue and the browning of white adipose tissue, contributing to anti-obesity effects.^(26)^ Therefore, the increased C14:1 level observed in this study may have contributed to reduced body weight gain in the coconut oil group, highlighting a potential physiological mechanism that warrants further investigation.

We also examined gene expression. Gene expression analysis revealed that the coconut oil group exhibited lower hepatic expression of *Scd1*, *Fasn*, and *Elovl6*, as well as reduced expression of the inflammatory marker *Tnfa*, compared to the lard group. *Scd1* is involved in hepatic lipid synthesis and β-oxidation, and *Scd1*-deficient mice have shown resistance to NAFLD.^(27)^ *Fasn* is a key enzyme in de novo lipogenesis and is upregulated in the livers of obese diabetic mice with NAFLD.^(28)^ *Elovl6* regulates fatty acid elongation and has been associated with insulin resistance and steatosis severity in NASH.^(29)^ Our findings suggest that MCFAs may suppress hepatic lipid synthesis and inflammation by downregulating these genes, thereby contributing to the prevention of hepatic fat accumulation.

This study is a pioneering investigation that used GC/MS for metabolomics analysis to examine the effects of MCFAs contained in coconut oil, compared to lard, on liver and glucose metabolism, and to elucidate the mechanisms by which MCFAs suppress fatty liver. However, the optimal dosage of coconut oil has not been sufficiently investigated in this experiment, and further research is required. Additionally, careful consideration is needed to determine whether the results from this animal study can be extrapolated to human health, and further clinical studies are necessary.

In conclusion, these findings suggest that coconut oil may contribute to the suppression of hepatic fat accumulation in the liver, and inhibition of weight gain established by a high-fat diet, by augmenting the levels of MCFAs in the liver and suppressing the gene expression related to inflammation and fat metabolism. Our study suggests that coconut oil consumption may play a pivotal role in preventing hepatic fat accumulation and could serve as a key element in the development of novel therapeutic strategies for NAFLD/MASLD.
